# Spinal Cord Stimulation for Intractable Chronic Limb Ischemia: A Narrative Review

**DOI:** 10.3390/jcdd11090260

**Published:** 2024-08-26

**Authors:** Roberto Gazzeri, Tommaso Castrucci, Matteo Luigi Giuseppe Leoni, Marco Mercieri, Felice Occhigrossi

**Affiliations:** 1 Interventional and Surgical Pain Management Unit, San Giovanni–Addolorata Hospital, Via Amba Aradam 9, 00184 Rome, Italy; 2 Vascular Surgery Unit, Sant’Eugenio Hospital, 00144 Rome, Italy; 3 Department of Medical and Surgical Sciences and Translational Medicine, Sapienza University of Rome, 29121 Rome, Italy

**Keywords:** intractable critical limb ischemia (ICLI), spinal cord stimulation (SCS), peripheral arterial disease (PAD), neuropathic pain, new SCS modalities, clinical outcomes

## Abstract

Critical limb ischemia (CLI) is the most severe form of peripheral arterial disease, significantly impacting quality of life, morbidity and mortality. Common complications include severe limb pain, walking difficulties, ulcerations and limb amputations. For cases of CLI where surgical or endovascular reconstruction is not possible or fails, spinal cord stimulation (SCS) may be a treatment option. Currently, SCS is primarily prescribed as a symptomatic treatment for painful symptoms. It is used to treat intractable pain arising from various disorders, such as neuropathic pain secondary to persistent spinal pain syndrome (PSPS) and painful diabetic neuropathy. Data regarding the effect of SCS in treating CLI are varied, with the mechanism of action of vasodilatation in the peripheral microcirculatory system not yet fully understood. This review focuses on the surgical technique, new modalities of SCS, the mechanisms of action of SCS in vascular diseases and the parameters for selecting CLI patients, along with the clinical outcomes and complications. SCS is a safe and effective surgical option in selected patients with CLI, where surgical or endovascular revascularization is not feasible.

## 1. Introduction

Peripheral arterial disease (PAD) of the lower limbs is one of the most common manifestations of atherosclerotic disease. Critical limb ischemia (CLI) is an advanced stage of PAD characterized by a significant reduction in arterial blood flow to the lower limbs. It is a pathological condition in which arterial disease results in chronic ischemic rest pain in the foot, often accompanied by ulcers or gangrene due to objectively proven arterial occlusive disease [1]. CLI is a chronic disorder corresponding to stages III and IV of Fontaine’s classification and stages 4, 5 and 6 of the Rutherford classification. The most important risk factors for the development of CLI are age, smoking and diabetes mellitus [2]. Diabetic patients are approximately ten times more likely to develop CLI requiring major amputation compared to non-diabetic PAD patients [3]. CLI is associated with high morbidity and mortality rates and is often poorly managed. The severe and often intolerable pain is caused by ischemia, areas of tissue loss and ischemic neuropathy. Ischemic rest pain commonly occurs at night, lasting from minutes to hours, and it is usually located in the distal part of the foot or near an ischemic ulcer or gangrenous toe. Pain often precedes the formation of an ischemic ulcer [4]. Furthermore, most patients with CLI present with comorbidities, such as diabetes, which increase the risk of complications and limb amputation.

The incidence of major amputations in CLI is typically 30–45% within one year, but this rate increases with concomitant diabetes as comorbidity [3,5,6]. The first-line treatment for CLI is revascularization, such as bypass surgery or angioplasty, which are essential for avoiding severe complications. However, patients with non-reconstructable CLI, such as those with previously failed bypass surgery, lacking suitable inflow or outflow vessels in selective angiography or the inability to undergo open surgery due to health status and associated comorbidities, may benefit from spinal cord stimulation (SCS), as an alternative approach, instead of conservative medical treatment [7]. SCS is an effective and safe treatment used for decades to reduce pain in conditions such as persistent spinal pain syndrome (PSPS) and complex regional pain syndrome (CRPS), based on the electrical stimulation of the dorsal columns of the spinal cord [8,9]. Given the opioid crisis and the often unsuccessful outcomes of conservative treatments, there is growing interest in utilizing SCS for patients with chronic pain [9,10]. Recent SCS devices offer substantially more advanced technological capabilities and waveforms compared to older-generation systems. The aim of SCS in CLI is not only to reduce pain but also to promote the trophic and functional recovery of the limb affected by ischemia. Rapid pain relief may also encourage patient mobilization. Achieving mobilization and reducing analgesic medication use can contribute to the training effects, which are part of the treatment goals of conservative PAD therapy. This review focuses on the mechanisms of action of SCS, the surgical technique, the parameters for selecting CLI patients, the new modalities of SCS in vascular diseases and clinical outcomes and complications.

## 2. Methods

**Figure 1:**
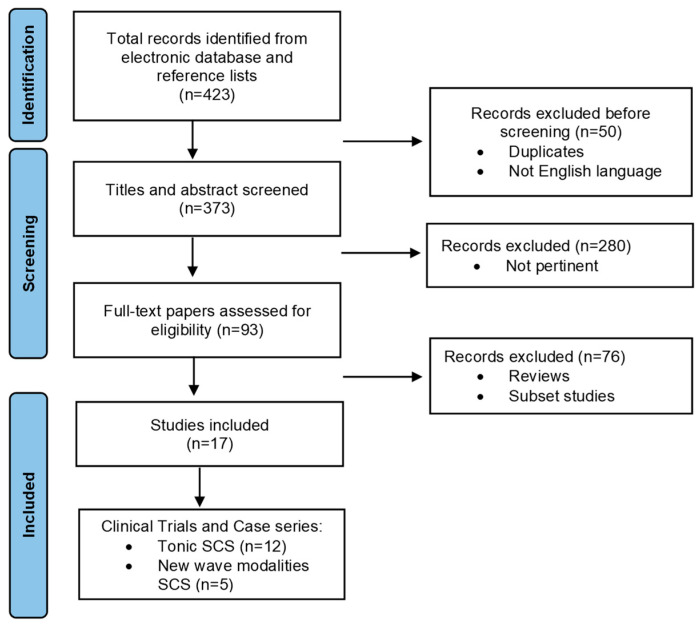
Flowchart of study selection process for SCS research. This flowchart illustrates the systematic selection process for clinical trials and case series assessing the efficacy of SCS in treating CLTI. From an initial pool of 423 records obtained from electronic databases and reference lists, 330 records were excluded because of irrelevance or lack of pertinence. The remaining 93 full-text articles were evaluated, leading to the exclusion of 76 articles due to reviews and subset studies. Finally, 17 clinical trials and case series were assessed for eligibility, with 12 studies focusing on tonic SCS and 5 exploring new wave modalities.

This literature research was conducted using electronic databases such as PubMed, Web of Science, Scopus and the Cochrane Library, with no limits on publication date. The following combinations of MESH terms and free text words were used for the search: “spinal cord stimulation” and “Intractable Critical Limb Ischemia (ICLI)”; “Peripheral Arterial Disease (PAD)” and “Peripheral Vascular disease” and “Amputation” or “Limb Survival”. Additional references were retrieved from the reference lists of articles available in the electronic database. A total of 423 articles were initially collected. Titles and abstracts were screened to identify articles eligible for further review. Rapid reviews, studies published in languages other than English, and reviews of laboratory or preclinical studies and case reports were excluded. Ninety-three full-text articles were evaluated after removing duplicates and subset studies, and 17 articles were finally included in this study. Of these, only 5 studies reported on the use of SCS with novel waveform modalities for the treatment of ICLI (Figure 1).

### 2.1. Mechanisms of Action of SCS

The mechanisms of action by which SCS can improve clinical outcomes in PAD are complex and not yet fully understood, but they are thought to be multifaceted. Various theories have been proposed, including the antidromic stimulation of small-diameter fibers, action on the spinal sympathetic nervous system and an increase in the level of inhibitory neurotransmitters [8]. Recent research suggests that the stimulation of sensory myelinated and unmyelinated fibers in the dorsal root ganglia by peridural electrodes activates cell-signaling molecules such as extracellular signal-regulated kinase and protein kinase. These kinases stimulate the transient receptor potential vanilloid receptor 1 (TRPV1), causing the release of vasodilators (e.g., calcitonin gene-related peptide), with microvascular effects [11]. Additionally, the subsequent release of endothelial nitric oxide (NO) further stimulates smooth muscle relaxation [12]. The final effect is a decrease in vascular resistance and an increase in local blood flow. Another mechanism of action for microcirculatory vasodilation includes the inhibition of autonomically mediated vasoconstriction through the inhibition of sympathetic neurotransmission at the ganglionic and post-ganglionic levels [13,14,15,16,17,18].

### 2.2. SCS Surgical

**Figure 2:**
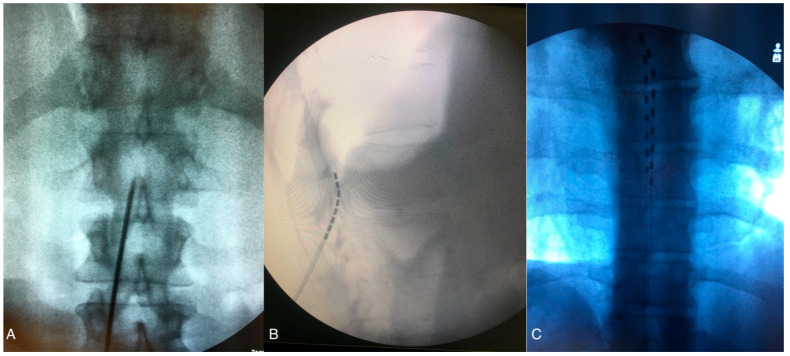
(**A**) Lumbar spinal X-ray, antero-posterior view: the Tuohy needle is advanced under fluoroscopy through the fascia toward the interlaminar space. (**B**) Fluoroscopic lateral view of the lumbar spine showing the tip of the needle entering the epidural space and being moved upward. (**C**) Antero–posterior fluoroscopic image of the two electrodes at T7 level. The figure was created by the authors.

Generally, patients undergo two-phase process for SCS. The first phase, known as the Trial Phase, is conducted to determine the optimal location for the implanted electrodes and to assess the rate of pain relief and patient satisfaction during the trial period. Patients who respond well during this phase are considered good candidates for permanent SCS implantation. During the Trial Phase, the procedure is performed on lightly sedated patients. With the patient in the prone position, real time radiological control is utilized throughout the procedure. After skin and subcutaneous tissue local anesthesia, a linear incision is made down to the lumbar fascia, usually between L2 and L5. The Tuohy needle is then advanced under fluoroscopy through the fascia toward the interlaminar space until the tip engages the ligamentum flavum, allowing access to the epidural space (Figure 2A). A low-resistance syringe filled with air or with saline is attached to the needle, and gentle pressure is applied while advancing the needle. When the tip of the needle enters the epidural space, a loss of resistance confirms peridural entry. The electrode is then advanced through the needle under fluoroscopic guidance and moved upward in the epidural space to the appropriate level (usually T7–T9) (Figure 2B,C). Anterior–posterior and lateral fluoroscopic views ensure the dorsal placement of the lead, and intraoperative paresthesia mapping confirms coverage of the painful areas. With the advent of new waveforms modalities, such as high-frequency or differential target multiplexed (DTM) SCS, electrode placement can be guided by radiological findings to ensure accurate positioning at the correct spinal level (mid T8 vertebra to mid T10) without the need for paresthesia mapping [19], as detailed in Paragraph 1.5. Once the leads are in place, they are anchored to the lumbar fascia. Extension leads are then connected to an external pulse generator (EPG), and a sterile dressing is applied. In SCS trials, success for chronic pain patients is primarily defined by achieving at least a 50% reduction in perceived pain, whereas, for CLI patients, trial success encompasses not only significant pain relief but also improvements in limb perfusion, wound healing, and limb salvage, highlighting the broader therapeutic goals beyond mere pain reduction. A second surgical phase is then performed to implant a permanent SCS. Under local anesthesia, the lead extensions are removed, and the permanent leads are tunneled subcutaneously to the pocket where the battery, or internal pulse generator (IPG), will be placed. Typically, the permanent IPG is located in the uppermost quadrant of the gluteal region.

### 2.3. Patient Selection: Inclusion and Exclusion Criteria for Spinal Cord Stimulation in CLI Patients

According to the recent literature, the selection of patients who would benefit from SCS therapy is crucial. Several selection criteria are reported in the literature, with the main inclusion criteria being patients with non-reconstructable CLI, those unable to undergo surgery due to health status or those who are poor candidates for interventional/endovascular approaches due to extensive vessel damage [20]. Other candidates include patients with multiple segmental arterial lesions of the lower extremities secondary to stenosis/occlusion of the ilio-femoral, femoro-popliteal or femoro-tibial arteries, as shown in selective angiography, the absence of autologous veins suitable for bypass graft surgery or those who had previously failed bypass surgery. For these patients, who have a low chance of undergoing reconstructive surgery, the inclusion criteria for SCS include persistent rest pain with or without skin lesions, a surface area of <3 cm^2^ in the feet and toes, and either the absence of ankle artery pulsation or incompressible ankle arteries [21]. Moreover, patient selection for SCS treatment is also based on radiological findings (ultrasound/angiography), which can determine the diagnosis of non-operable chronic CLI. Other possible selection criteria include lower limb skin microcirculation parameters, such as capillary microscopy and laser doppler. Recently, a fundamental parameter has gained importance in selecting appropriate candidates for SCS therapy: the transcutaneous oxygen tension measurement (TcPO_2_). The TcPO_2_ assesses skin oxygen supply by the superficial microvascular system, with a TcpO_2_ <30 mmHg indicating CLI, as stated by the Transatlantic Inter-Society Consensus [1]. TcPO_2_ measurements have been preferred by several authors as a non-invasive method for assessing local skin microcirculation and as parameter to predict ulcer healing or limb amputation. To assess the functional state of the peripheral vascular perfusion, an orthostatic test is performed (from lying to standing position), while the measure of resting foot skin perfusion (resting TcPO_2_) is assessed with the patient in lying position.

Although baseline TcPO_2_ is extremely important to achieve good clinical outcomes, TcPO_2_ measurements are technically challenging and time-consuming [7]. Claeys and Horsch [22] observed significant pain relief during SCS in patients with preoperative TcPO_2_ assessment. After SCS implantation in CLI patients, the probability of limb survival increases for patients selected based on the TcPO_2_ measurement. Ubbink et al. [23] reported a limb survival probability of 77% in patients with intermediate baseline TcPO_2_. In Amann et al.’s study, patients without SCS had only a 45% probability of limb survival, while in SCS-treated patients, it was 78% for those with TcPO_2_ > 30 mmHg at baseline and TcPO_2_ > 10 mmHg at baseline, rising to 20 mmHg after the SCS trial [6]. When the TcPO_2_ levels < 10 mmHg, there is a higher risk of amputation due to depleted microcirculation capacity, whereas TcPO_2_ levels > 30 mmHg indicate that the patients’ clinical conditions are likely to improve with SCS implantation. During the SCS trial test, an increase in TcPO_2_ > 10 mmHg suggests the possibility of permanent SCS implantation for limb salvage [6]. Kumar et al. observed that patients with TcPO_2_ < 10 mmHg after SCS tended to undergo early limb amputation [24]. Klinkova et al. noted that an increase of >10 mmHg in standing TcPO_2_ during orthostasis was associated with an increased probability of a positive clinical outcome after SCS, while chronic morphofunctional changes in the microcirculatory system, indicated by a resting TcPO_2_ less than 10 mmHg and the absence of TcPO_2_ levels in orthostasis, negatively affected the outcome [21]. Moreover, the same authors reported an increase in resting TcPO_2_ values to 40 mmHg in the affected foot in more than 70% of the patients with SCS [21].

To better understand the impact of SCS on perfusion indices in patients with CLI, we summarized the physiological changes associated with SCS therapy in Table 1. SCS has been shown to improve various measures of blood flow and tissue oxygenation, which are critical for assessing the therapeutic success and potential limb salvage in CLI patients. The following table summarizes the key effects of SCS on several perfusion indices, including TcPO_2_, ankle-brachial index (ABI), skin perfusion pressure (SPP) and capillary blood flow. These indices provide valuable insights into the mechanisms by which SCS may contribute to pain relief, enhanced wound healing and overall clinical improvement in this patient population.

The exclusion criteria for performing SCS reported in the literature include intermittent claudication with no rest pain, skin lesions with ulceration and gangrene larger than 3 cm^2^ and deeper than muscle fascia, infection of the skin lesions, gangrene in more than one toe, extensive non-healing ulcerations, patients with concomitant disease with a life expectancy less than 1 year, the impossibility of implanting the electrode in the epidural space of the spinal canal and patients with inadequate compliance due to psychosocial difficulties. Another exclusion criterion is a reduction in pain of less than 50% during the trial SCS phase; in these cases, the permanent implantation of the SCS is not recommended [24].

### 2.4. Spinal Cord Stimulation and CLI

Within the past decade, SCS has been proposed for the management of patients with CLI who cannot be treated with endovascular approaches or open bypass surgery. SCS plays an important role in treating non-reconstructable CLI. Although SCS therapy was introduced in the 1960s for chronic pain treatment, Cook et al. were the first to describe the use of SCS in PAD in 1976. They found an improvement in pain relief and ulcer healing with SCS treatment in a patient with painful ischemic ulcers in lower extremities [29]. Claeys and Horsh compared SCS to the medical treatment with prostaglandin in 231 CLI cases (111 vs. 120), reporting significantly better healing of foot ulcers in the SCS group (69% vs. 17%) [22]. Interestingly, foot TcPO_2_ increased significantly in the surgical group. In the Belgian study of Suy et al., 20 CLI patients with SCS implants were compared to 18 cases with conservative treatment [30]. The implant group showed better clinical outcomes in terms of relief of ischemic rest pain, walking distance, social life and the healing of ulcers. Jivegard et al. evaluated the effect of SCS in patients with non-operable severe leg ischemia compared to analgesic treatment alone, observing long-term pain relief only in the SCS group [31]. Although the rates of limb salvage were similar in the two groups, the extent of amputation was smaller in the SCS patients. The results of the European Peripheral Vascular Disease Outcome Study (SCS-EPOS) by Amann et al. showed a 78% limb survival in non-reconstructable patients, along with a substantial decrease in the percentage of patients using narcotic analgesics, from 58% at baseline to 29% during SCS treatment [6]. Reig and colleagues evaluated 98 patients with PAD treated with SCS, reporting that 88% of cases experienced good pain relief [14]. Klomp et al., in the ESES study, reported a hazard ratio for 2-year survival without major amputation in the SCS group compared to the standard group of 0.96 (95% CI: 0.28–1.28) [32]. Furthermore, the ESES study observed a linear improvement in patient-reported outcome measures (PROMs) in 60 patients who underwent SCS plus medical treatment, with better mobility and energy scores compared to the control medical treatment group [33].

In 2013, the Cochrane review analyzed data from 433 patients in six RCTs investigating the outcomes of SCS in critical limb-threatening ischemia (CLTI) [34]. Three of these RCTs were from the ESES study, each addressing a different endpoint or aspect [23,32,33]. The primary endpoint of all studies was limb salvage. Limb salvage rates were significantly higher in the SCS group compared to conservative medical treatment. The results were better when the patients were selected based on their initial TcPO_2_. Significant pain relief was found in both treatment groups, but the SCS group required fewer analgesics. The overall complication rate was 17%, with implantation problems occurring in 9% of cases. The study confirmed that SCS had a modest positive effect on pain relief, with an 11% reduction in the amputation rate at 12 months, compared to conservative management [23]. In the retrospective controlled study by Liu et al., the VAS score in the SCS treatment group improved significantly one week and one year after treatment, from 8.63 to 2.5, respectively. Additionally, lower limb scintigraphy revealed an increase in microcirculation intensity in the lower extremities [35]. The retrospective analysis by Tshomba demonstrated functional clinical success in 25.7% of 101 patients, with major and minor amputations occurring in 5.9% and 6.9% of cases, respectively [36]. In a retrospective study using tonic stimulation (PrimeAdvanced SureScan Medtronic, Minneapolis, MN, USA) for neuromodulation, Cucuruz et al. reported an 88% limb salvage rate, with a significant reduction in pain on the 10-point VAS scale from baseline (median = 7.5) to follow-up at 2 years (median = 0), *p* < 0.001. Walking distance also improved from a median of 50 m preoperatively to 150 m at the 2-year follow-up [37]. In their series of 56 CLI patients implanted with the Eon mini rechargeable generator (St. Jude Medical Inc, Chicago, IL, USA), Klinkova et al. reported that more than 70% of cases showed varying degrees of positive outcomes: 88% of patients had no more leg pain at rest and experienced an improvement in painless walking distance [21]. In 20% of patients, complete healing of ulcerative foot defect was noted. Limb salvage at 1-year follow-up was 96.2%, although two patients underwent major amputation. In the first case of CLI with associated diabetic peripheral polyneuropathy, the resting TcPO_2_ was 9 mmHg, and standing TcPO_2_ did not increase in the orthostatic position, confirming irreversible ischemic changes of the microvasculature. The other case had a resting TcPO_2_ of 5 mmHg that did not increase with orthostasis. Cyrek et al. reported 29 CLI patients who underwent implantation with the Genesis generator (St. Jude Medical Inc., USA) [38]. At the 3-month follow-up, pain intensity on the VAS decreased from 8.1 to 4.5. At 6 months, a further decrease was observed (VAS 2.3), while at 24 months, the final VAS was 1.6. At the 3-month follow-up, all patients with Fontaine stage III improved to Fontaine stage II or I. The amputation rate was 3% (1 patient with Fontaine stage IV), with no mortality in the group. No differences were observed between diabetic and non-diabetic patients in outcome, but the non-diabetic group showed a significantly lower VAS score at 12 months. In a retrospective study published in 2023, Piedade et al. analyzed the results of 72 patients with a CLI diagnosis (stage III and IV according to Fontaine’s classification) who underwent SCS implantation (66 Genesis, 5 Eon; St. Jude Medical/Abbot) [7]. In their series, they reported a higher probability of limb survival at 12 months after SCS implantation in Fontaine’s stage III (94%) compared to stage IV (62%). Major amputations occurred more slowly in Fontaine’s stage III (19.0 months vs. 19.8 months). These findings have important clinical implications for the management and treatment of CLI patients. Specifically, the data suggest that early intervention in patients with less advanced disease may prolong limb survival and delay the need for major amputation.

### 2.5. Novel Neurostimulation Modalities and Waveforms in Chronic Pain

SCS has been used for several decades to treat chronic neuropathic pain. Typically, SCS activates the dorsal columns of the thoracic spinal cord. Conventional SCS (tonic stimulation) is believed to depolarize the Aβ fibers of dorsal columns in both antidromic and orthodromic directions. In the antidromic direction, SCS can activate GABAergic inhibitory interneurons, which inhibit the incoming signals from nociceptors and interfere with further processing of the nociceptive signal through the spinothalamic tract. In orthodromic direction, SCS can depolarize Aβ fibers cranially, modulating the supraspinal centers [35,39]. SCS waveforms are characterized by different frequency, pulse widths (durations), amplitudes and pulse shapes. Depending on the stimulation waveform and modality, different nervous system cell types, such as neural cells and astrocytes, may be activated, or the inhibitory pathway may be engaged, resulting in spinal cord neurostimulation. In the past decades, conventional tonic SCS relied on inducing a sense of paresthesia that had to cover the area of pain to provide pain relief. Conventional tonic SCS involves repetitive low frequency pulses (50–100 Hz), high pulse width (200–500 ms) and high amplitude (3.5–8.5 mA), which cause paresthesias over the area of pain. Recently, novel paresthesia-free waveforms, including high frequency, burst and differential target multiplexed (DTM) stimulation, have been developed for treating chronic neuropathic pain [17]. These new waveforms do not rely on generating paresthesias for efficacy, unlike traditional SCS. Current evidence suggests that these new SCS modalities are effective for treating chronic pain and appear superior to classical tonic SCS. While tonic, burst and high frequency waveforms focus on stimulating neural cells located in the dorsal horns of the spinal cord, DTM targets the supportive glial cells in the nervous system by modulating dorsal horn glial gene expression [40,41]. Glial cells, predominant at the neurostimulation site, contribute to chronic pain mechanisms by responding to neural distress signals with the release of protective and pathological signaling molecules and inflammatory signals, increasing the sensitivity of pain signaling neurons.

DTM-SCS (Medtronic, Minneapolis, MN, USA) uses a classical waveform, characterized by multiplexed signals with varying frequencies (50 Hz to 1200 Hz), amplitude and pulse width (50 to 400 ms). The first report on DTM stimulation was published in 2020, showing promising preclinical results. Furthermore, Fishman et al.’s multicenter observational study described significantly better pain relief in patients with DTM SCS compared to conventional tonic SCS [42].

Burst waveform stimulation (St. Jude Medical-Abbot, Plano, TX, USA) was developed by De Ridder and is characterized by a frequency of 40 Hz, with a pulse width of 1000 μs, consisting of spikes with an intraburst frequency of 500 Hz and a quiescent phase between the burst cycles, with a 1 mA amplitude. Several studies have compared burst SCS and high frequency SCS with conventional low-frequency SCS for the treatment of various chronic pain conditions. In a series of 100 patients with chronic limb and back pain, burst SCS was found to be safer and more effective than conventional SCS, as evidenced by a significant decrease in VAS scores over a 1-year follow-up period [13].

Other studies comparing burst SCS with sham stimulation (turning the stimulation amplitude to zero) found better pain relief with burst stimulation [43,44].

High frequency stimulation (Nevro Corp., Redwood City, CA, USA) is characterized by high frequency (10 kHz), with a low pulse width (30 μs) and low amplitude (1–5 mA). This 10 kHz SCS results in subthreshold stimulation without paresthesias, eliminating the need for intraoperative mapping of paresthesias, with electrodes placed anatomically, based on the pain area [45]. The mechanism involved in the paresthesia-free 10 kHz SCS relies on a selective activation of inhibitory interneurons in the dorsal horn of the spinal cord, sparing dorsal column fibers activation [46]. In a study comparing 10 kHz HF-SCS with conventional tonic SCS in 198 patients with chronic back and leg pain, Kapural et al. reported that more patients responded to HF10 kHz-SCS, showing a greater decrease in VAS scores [45].

WaveWriter technology’s Fast-Acting Sub-Perception Therapy (Boston Scientific Corporation, Valencia, CA, USA) uses sub-perception stimulation with paresthesia-based low-frequency stimulation using multiple waveforms simultaneously. This approach enables tailored neurostimulation patterns based on patients’ needs. The first multicenter observational case series study using sub-perception SCS methodology reported significant pain relief, with rapid onset, and a reduction of 7.1 points on the VAS scale [47].

### 2.6. Novel Neurostimulation Modalities and CLI

SCS with new waveform paradigms offers a promising therapeutic option for patients with CLI and limb pain. However, there are only a few studies or case reports that have investigated the efficacy of new waveforms for patients with non-operable CLI. The 2021 systematic review by Asimakidou and Matis found no studies comparing new waveform SCS to conservative treatment in CLI patients [48]. In a study by Ueno et al., involving patients with unbearable leg pain secondary to CLTI, who underwent implantation of an SCS system (Intellis IPG Medtronic), 18 out of 20 patients received traditional low-dose therapy, while two cases used new waveform modalities: one patient required high-dose therapy, and the other underwent DTM SCS [49]. The authors suggest using more suitable paradigms, such as DTM and high-dose therapy, for CLTI patients with leg pain, noting that battery charging is more frequent compared to low-dose therapy. They also emphasize that preserving the microcirculatory vascular bed and maintaining the hemoglobin levels above 11.4 g/dL are fundamental for SCS efficacy in CLTI patients [49].

Kilchukov et al. reported a comparative advantage of HF-SCS over low-frequency SCS (LF-SCS) at 3 months, with a mean VAS score of 2.8 and 3.3, respectively, with this clinical improvement persisting at the 12-month follow-up. Various SCS systems with different stimulation parameters were used, including 31 Precision spectra (Boston Scientific Corporation, Valencia, CA, USA), 7 Proclaim XR (Abbot, Plano, TX, USA), 1 Restore Sensor SureScan MRI (Medtronic, Minneapolis, MN, USA), 11 Freedom 8A (Stimwave, Pompano Beach, FL, USA) [26]. In 25 patients, new waveform parameters (HF-SCS) were used, while in the other 25 cases, LF-SCS parameters were used. HF-SCS provided a greater decrease in calf pain severity at both 3-month and 12-months follow-ups. Although no intergroup difference in TcPO_2_ was observed, one patient required amputation 10 months after LF-SCS implantation. In a long-term retrospective study, Kretzschmar et al. evaluated survival and amputation outcomes in 49 patients treated with SCS using various devices, including 42 with tonic stimulation and 7 with paresthesia-free passive recharge burst stimulation (Eon, Eon mini, Genesis, Proclaim, Proclaim XR, Prodigy-MRI; St. Jude Medical/Abbot, Plano TX, USA) [50]. The patients showed a 91% reduction in NRS scores, from 7.7 at baseline to 0.7 at 24 months follow-up. Among the 30 patients on opioid medications, 63% reported no opioid usage at the 2-year follow-up. None of the 7 cases with recharge burst stimulation required IPG replacement [50]. De Caridi et al. utilized new waveform modalities of SCS as an adjuvant treatment in 34 cases of severe PAD (severe ischemic pain, ischemic lesions, late bypass failure and early failure of multiple endovascular and surgical revascularization). In their series, the impulse generators used included the Nevro Senza with a 10 kHz SCS System, as well as devices from Medtronic and St. Jude Medical. The results of their study were very encouraging, with healing rates ranging from 57% to 100% [51]. A recent study by Ouerchefani et al., presented at the International Neuromodulation Society Conference in 2024, reviewed the results of 53 patients diagnosed with CLTI and treated with SCS. New sub-perceptive waveforms (Boston Scientific Corporation, Valencia, CA, USA) were used in 38.5% of cases. The cumulative incidence of major amputation at the 1-year follow-up showed a better outcome compared to the natural history of the disease (13% vs. 30–45%). In their series, 85% of cases were free of major amputation at the 2-year follow-up [28]. Table 2 summarizes the characteristics of studies on new waveforms in SCS.

### 2.7. Exploring the Necessity of SCS Trials in the Treatment of CLI Critical Limb Ischemia

Recently, the use of trials during SCS has been questioned due to a possible cost reduction and reduced complication by adopting an implantation strategy without a screening trial [52,53]. Consequently, PAD patients with CLI are potentially the best candidates for an all-in-one SCS implantation strategy. However, further studies are required to confirm these hypotheses. In our clinical practice, we normally conduct an extended trial period of nearly four weeks to thoroughly evaluate the outcomes of SCS beyond mere pain relief. This trial is essential in assessing several primary objectives that are critical for determining the therapy’s overall success. Patient satisfaction is a key component of the trial evaluation, as it provides valuable insight into whether patients experience significant improvements in their quality of life, mobility and daily functioning. These factors are crucial indicators of therapy success, reflecting how well the treatment aligns with patients’ expectations and enhances their daily lives. Additionally, monitoring the initial healing of tissue and improved limb perfusion during the trial is vital for assessing the effectiveness of SCS in addressing CLI. Observing early signs of tissue healing can indicate positive physiological responses to the treatment and provide an early indication of potential long-term benefits. Economic considerations also play a significant role in the trial process. Given the substantial cost associated with SCS procedures, it is important to conduct a thorough trial to ensure that only patients who are likely to benefit proceed to permanent implantation. This careful evaluation helps to reduce the risk of financial waste by confirming the therapy’s efficacy and appropriateness for each patient before committing to a costly long-term treatment plan. In summary, we believe that the extended trial period serves as a comprehensive evaluation of SCS outcomes, focusing on patient satisfaction, initial physiological improvements and economic viability. By considering these factors, we aim to optimize patient selection and ensure that the therapy provides meaningful and lasting benefits.

## 3. Complications

The risk of complications in SCS surgery ranges from 1.4% to more than 20% of cases [54,55]. Anatomical challenges and technical issues can lead to difficulties in electrode placement within the epidural space. According to a review by Ubbink and Vermuelen, the risk of potential adverse events was 8%, with lead dislocation or fracture occurring in 12% of cases [23]. Amman reported a 2.6% failure rate for electrode positioning [6], while Spincemaille documented a complication rate of 15% due to malpositioning [55]. In their review of SCS complications, Eldabe et al. reported lead migration or lead fracture/disfunction in 21.86% of cases, with battery failure occurring in 1.7% of patients [26]. More recently, Klinkova reported electrode migration in 20.4% of patients between 4 and 10 months after SCS implantation [21]. In the series of Kilchukov et al., one case of electrode migration required surgical revision [27].

CLI patients generally have a higher risk of infection compared to the general population due to compromised blood flow and poor wound healing, associated with ischemia. The presence of ulcers or gangrene in these patients may further elevate the risk of infection following SCS implantation. In particular, surgical site infections may occur more frequently in CLI patients due to these factors. Infection of the lead implant site or subcutaneous IPG pocket are serious complications that may require explantation of the SCS implant. The Cochrane review reported a 3% infection risk at the lead site or subcutaneous IPG pocket [56], while Eldabe et al. found a 4.89% rate of deep and superficial wound infections [26]. De Caridi et al. reported four SCS removals in their series of 34 patients: two removals were due to local infection, and two others were removed for malfunction in Rutherford Class 6 cases. All four patients underwent lower limb amputation [51]. Recently, Piedade encountered a severe infection in one patient (1.4%) after the trial phase. Despite one-quarter of their patients being diabetic, no infections occurred in that subgroup [7]. The trial phase of neuromodulation with external leads may increase the risk of infection, especially in patients with compromised immune systems or diabetes. This phase requires a second surgery to implant the IPG subcutaneously. However, it is a necessary step because if the test stimulation during the trial phase results in less than 50% pain reduction and functional improvement from the baseline, permanent implantation should be avoided. Less frequent complications after SCS implantation include epidural hematoma (0.3%), dural puncture (0.3%) and skin erosion (0.2%) [25,56]. The SCS trial and permanent implant procedures carry a high-risk of bleeding, putting the patient at risk for epidural hematoma. As most patients affected by CLI are on antiplatelet therapy, the slight increase in bleeding risk related to the procedure should be weighed against the general cardiovascular risks of suspending the therapy [57].

## 4. Future Directions

Despite the advancements in SCS technologies and techniques, the current body of literature on SCS for CLI remains limited in scope, particularly in the context of randomized clinical trials. This highlights a significant gap in the existing research, which necessitates further investigation to establish evidence-based practices. Moreover, as novel waveform modalities and technologies in SCS continue to evolve, it is crucial to evaluate their long-term outcomes across diverse patient populations in the future. Furthermore, the variability in patient selection criteria, study designs and outcome measures in the current literature underscores the need for standardized methodologies. The potential benefits of SCS, such as pain relief, improved limb perfusion and enhanced quality of life, underscore the importance of ongoing research to maximize patient outcomes. By filling the gaps in our current understanding, future research can refine therapeutic strategies, minimize complications, and ultimately enhance the efficacy of SCS for patients with CLI. Future research and more randomized controlled trials (RCTs) are necessary to assess the effects and mechanisms of novel SCS waveforms and their establishment in clinical practice. Early SCS implant in PAD patients may help maximize the benefits of this surgical treatment.

## 5. Conclusions

SCS for CLI has shown promising results, but there remain significant unmet needs and future directions that require attention. There is a critical need to establish clear guidelines and indications for SCS in the management of ICLI, as this could significantly enhance patient outcomes. Our review shows that patients with higher baseline TcPO_2_ levels, or those who experience an increase in the TcPO_2_ after SCS, tend to have better clinical outcomes. We suggest that there is a pressing need to integrate the TcPO2 as a standardized biomarker for SCS candidacy. By incorporating the TcPO_2_ measurements into patient selection criteria, clinicians can better identify individuals who are more likely to benefit from SCS, ensuring timely and effective intervention. This approach could prevent delays in treatment, allowing for earlier intervention, which could halt or even reverse disease progression. Implementing SCS at an earlier stage, before significant tissue damage occurs, could enhance limb perfusion, facilitate wound healing and ultimately reduce the likelihood of major amputations. Shifting the focus of SCS from a last-resort salvage therapy to an early intervention strategy represents a significant advancement in CLI management, promising to improve patient outcomes and quality of life.

Novel SCS waveforms offer an alternative therapeutic option for non-operable patients with ICLI. While traditional tonic SCS has demonstrated beneficial effects, the magnitude of these effects has been relatively small. However, SCS with new waveforms paradigms presents a promising therapeutic option for these complex patients. In our review, we considered the criteria for identifying the right candidates and evaluated the safety of the novel SCS devices, as well as patients’ satisfaction regarding pain relief, quality of life and clinical improvement. SCS is still underutilized in CLI patients, and more studies are needed to evaluate the efficacy and safety of new SCS waveforms.

## Figures and Tables

**Table 1:** Concise overview of how SCS affects various perfusion indices, highlighting its potential benefits and variability in outcomes. The studies used in this table are among the 17 studies finally selected for this review.

Perfusion Index	Effect of SCS	Description	References
Transcutaneous Oxygen Pressure (TcPO_2_)	Increase in TcPO_2_ Levels	SCS has been shown to significantly increase TcPO_2_ levels in CLI patients, indicating improved microcirculation and oxygenation. Higher baseline TcPO_2_ and increases post-SCS correlate with better limb salvage outcomes.	Horsch and Claeys [22] (1996); Amann et al. [6] (1984); Kumar et al. [25] (1997)
Ankle-Brachial Index (ABI)	Variable Improvements	While SCS may improve ABI in some patients, results are inconsistent. Improvements are more likely in patients with initially higher TcPO_2_ levels.	Horsch and Claeys [22] (1996); Ubbink et al. [23] (1999)
Skin Perfusion Pressure (SPP)	Enhancement	SCS enhances SPP by promoting vasodilation and increasing blood flow to ischemic regions, potentially improving wound healing.	Eldabe et al. [26] (2010); Klinkova et al. [21] (2015)
Capillary Blood Flow	Increased Flow	SCS may improve capillary blood flow by reducing vasoconstriction and stimulating vasodilator release, contributing to pain relief and ulcer healing.	Kilchukov et al. [27] (2022)
Resting TcPO_2_ and Orthostatic TcPO_2_	Significant Rise in Orthostatic TcPO_2_	SCS often leads to a marked increase in orthostatic TcPO_2_, which is associated with positive clinical outcomes and reduced amputation risk.	Reig et al. [14] (1991); Ouerchefani et al. [28] (2024)

**Table 2:** Characteristics of new waveform SCS studies.

References	N° pts	Study Characteristics	Disease Type	SCS Device	Follow Up	Pain Relief	Limb Salvage	Complications
De Caridi [51] (2016)	34	Observational Study	PAD; CLI	Nevro Senza; Medtronic; St. Jude Medical	12 mths	From 57% to 100% of pain relief	4 cases of limb amputation	1 infection 2 removals for wound dehiscence
Kilchukov [26] (2023)	50	RCT Randomized Clinical Trial	CLTI	Boston Scientific, Precision spectra: 31 cases; Abbot, Proclaim XR: 7 cases; Medtronic, Restore Sensor SureScan MRI: 1 case; Stimwave, Freedom 8A: 11 cases	12 mths	Preop HF-SCS: 7.8 LF-SCS:8.1 Postop HF-SCS 2.8 LF-SCS 3.3	98% (1 limb amputation with LF-SCS)	1 infection (LF-SCS) 1 lead migration (LF-SCS)
Kretzman [50] (2023)	49	Retrospective Study	PAD	St. Jude Medical/Abbott	48 mths	Preop: 7.7 Postop:0.40	89.8% (5 cases of limb amputation)	9 lead replacement
Ueno [49] (2024)	20	Retrospective Study	CLTI, SALI	Medtronic Intellis: 18 LF, 1 HF, 1 DTM	17 ± 14 mths	Preop: 10 Postop: 4	100%	1 infection
Ouerchefani [28] (2024)	53	Observational Case Series Study	CLTI	Boston Scientific 32 LF; 20 FAST	24 mths	Preop: 9.4 Postop: 3.7	85% (5 limb amputations, Fontaine stage IV)	None

CLTI: chronic limb-threatening ischemia; ICLI: intractable critical limb ischemia; CLI: critical limb ischemia; SALI: subacute limb ischemia; PAD: peripheral artery disease; mths: months; SCS: spinal cord stimulation; LF: low frequency; HF: high frequency.

## Abbreviations

CLI	critical limb ischemia
CLTI	chronic limb-threatening ischemia
SCS	spinal cord stimulation
DTM	differential target multiplexed
FBSS	failed back surgery syndrome
PAD	peripheral artery disease
TcPO_2_	transcutaneous oxygen tension
